# Slipped capital femoral epiphysis with hypopituitarism in adults: A case report and literature review

**DOI:** 10.1097/MD.0000000000028256

**Published:** 2021-12-23

**Authors:** Zhixin Niu, Jinshuo Tang, Xianyue Shen, Shenghao Xu, Zhongsheng Zhou, Tong Liu, Jianlin Zuo

**Affiliations:** a Department of Orthopedics, China-Japan Union Hospital of Jilin University, Changchun, China; b Department of Orthopedics, The Second Hospital of Jilin University, Changchun, China.

**Keywords:** adult slipped capital femoral epiphysis, case report, congenital hypopituitarism, review, total hip arthroplasty

## Abstract

**Rationale:**

Slipped capital femoral epiphysis (SCFE) is a common disease in pediatric orthopedics. Most research on SCFE has focused on high-risk groups or the whole population, and studies focusing on adult SCFE patients are rare. In the present study, we report the case of an adult patient with SCFE.

**Patient Concern:**

A 37-year-old man presented to our clinic with persistent pain that was poorly localized to both hips, groin regions, and thighs for more than 1 year.

**Diagnoses:**

A bilateral hip X-ray examination was performed, and the femoral epiphyses were found to be unfused on both sides. Low levels of growth hormone (GH), insulin-like growth factor-1 (IGF-1), triiodothyronine (T3), thyroxine (T4), follicle-stimulating hormone, luteinizing hormone, estradiol, and testosterone, and high levels of thyroid-stimulating hormone, prolactin, and cortisol.

**Interventions:**

Hormone-substitution therapies (levothyroxine sodium to treat hypothyroidism and testosterone enanthate to treat hypogonadism) were prescribed. Total hip arthroplasty was performed to treat femoral epiphysis slippage.

**Outcomes:**

After 6 months of postoperative follow-up, the patient's gait improved significantly, and bilateral hip pain was relieved.

**Lessons:**

When treating adults with SCFE, clinicians must be alert to endocrine disorders. Comprehensive imaging evaluation is crucial for the accurate diagnosis and selection of an appropriate treatment.

## 1 Introduction

The incidence of slipped capital femoral epiphysis (SCFE) is extremely low in the general population, and significant differences are observed across regions, ethnic groups, sexes, and age groups. The risk factors that cause this disease are complicated and include obesity, growth spurts, and in some rare cases, endocrine disorders. For example, epidemiological studies have shown that the incidence of SCFE is 10.8/100,000 in the United States, whereas in Japan, the SCFE incidence rates are 2.22/100,000 and 0.76/100,000 among boys and girls, respectively, at the ages of 10–14 years.^[1,2]^ Endocrine abnormalities, such as hypothyroidism, hypogonadism, and panhypopituitarism, should be considered if patients are younger than 8 years or older than 15 years.^[3]^

Macía-Villa et al^[4]^ were the first to review all published cases of SCFE in adults. With the development of medical technology, novel diagnostic and treatment methods have been increasingly used in clinical practice in recent years. Here, we review all case reports of SCFE in adults published before January 2021 and summarize the research progress made in diagnosis, treatment, among others. Furthermore, we describe the case of a 37-year-old man with adult-onset SCFE associated with multiple pituitary hormone deficiencies.

## 2 Case presentation

**Figure 1:**
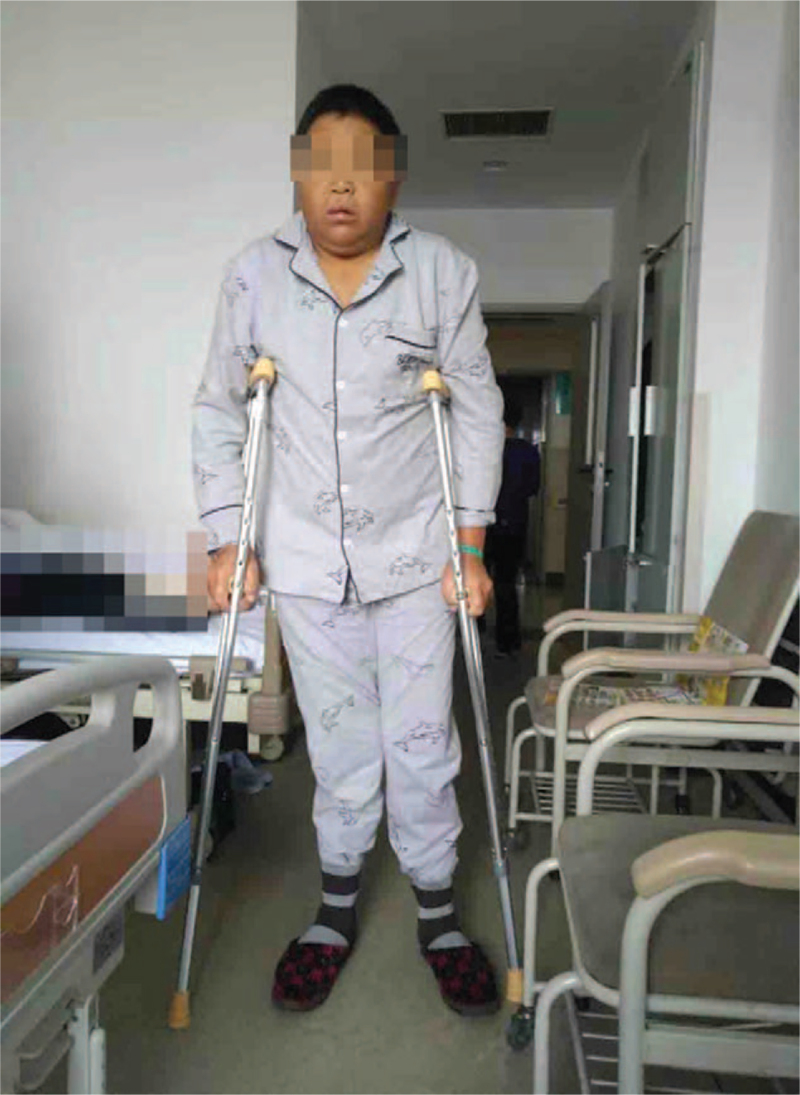
The patient cannot stand without crutches.

**Figure 2:**
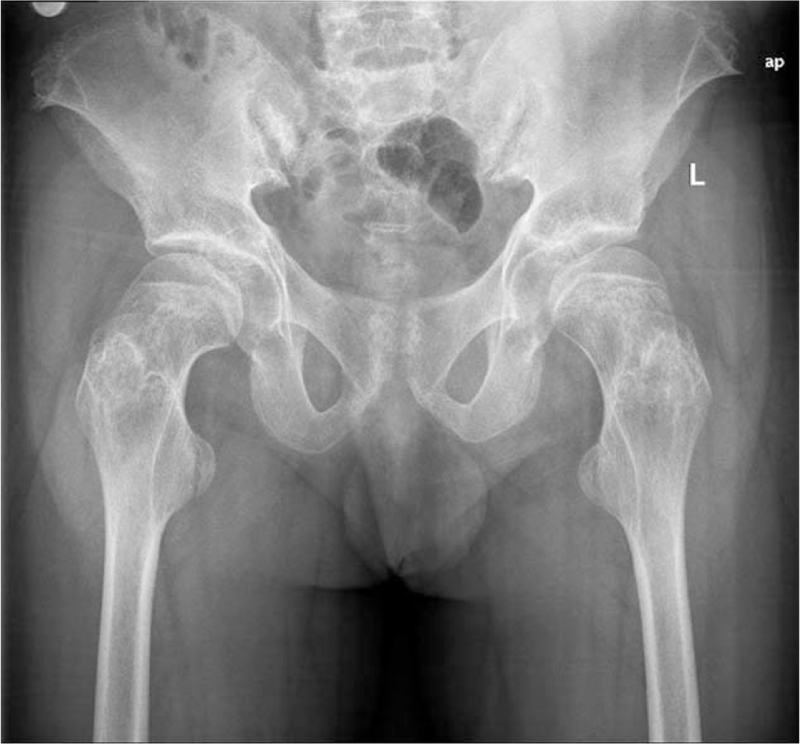
Anteroposterior radiograph of the hips demonstrates bilateral unfused femoral proximal epiphyses and ossification centers of the lesser trochanters.

**Figure 3:**
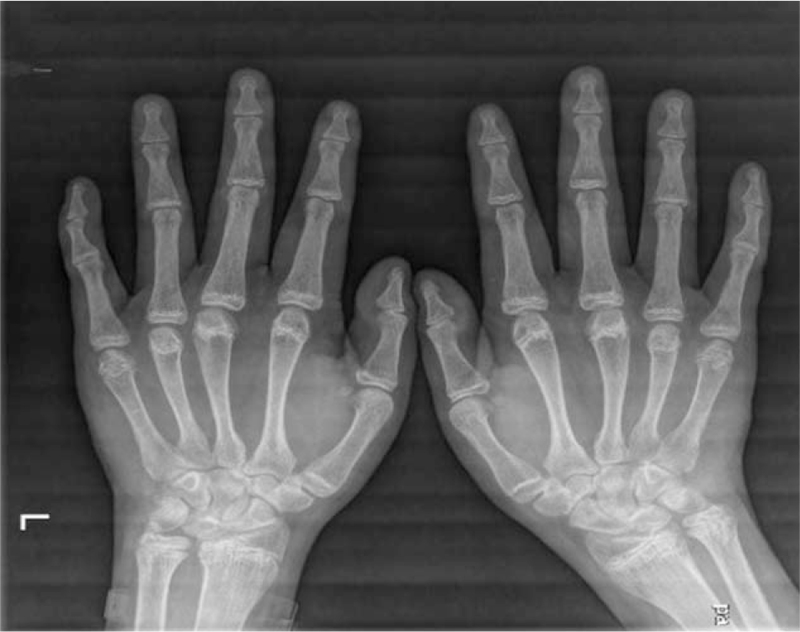
x-Ray examination of both hands demonstrates that the bone age of the patient is 16 years.

This study was approved by the Ethics Committee of the China-Japan Union Hospital of Jilin University. Signed informed consent was obtained from all patients in accordance with the Declaration of Helsinki. The patient was born in 1981 and presented to our clinic with persistent pain that was poorly localized to both hips, groin regions, and thighs for >1 year. He underwent an evaluation at a local hospital and was administered analgesics. In the past 3 months, the pain in the left hip had worsened, so he could not ambulate flexibly and had to use crutches (Fig. 1). The patient was referred to our hospital for diagnosis and further treatment. We performed a bilateral hip x-ray examination and found that the femoral epiphyses were unfused on both sides (Fig. 2). Further x-ray examination of both hands (Fig. 3) revealed bilaterally unfused epiphyses in the phalanges, metacarpals, proximal ulna, and radius. The patient's bone age was only 16 years. A detailed physical examination revealed bilateral groin tenderness, limited range of motion in all directions in both hips (with the limitation being more severe in the left hip), a positive Patrick sign in the left hip, a Harris hip score (HHS) of 40, a visual analog scale (VAS) score of 3, immature genitals and pubic hair (Tanner stage II), and absence of the prominentia laryngea. According to his family members, his mother once took an oral contraceptive pill while she was pregnant with the patient, but he was born full term. During childhood and puberty, he was shorter in height than his peers, and his growth velocity was slower. At the age of 15 years, the patient's height was approximately 130 cm, and his physical growth and development were arrested until the age of 30 years. From the age of 30 onwards, his height began to increase at a rate of approximately 3 cm/year, without any apparent triggers. This growth velocity has decreased over the past 3 years. At the time of admission to our hospital, his height was 169 cm. His weight did not change significantly over the past 3 months.

The medical history of delayed development of the stature of the patient raised suspicion of an endocrine disorder, and he was referred to the endocrinology department for further assessment. Hormone levels were evaluated because of abnormal height and delayed puberty, and the results revealed low levels of growth hormone (GH), insulin-like growth factor-1 (IGF-1), triiodothyronine (T3), thyroxine (T4), follicle-stimulating hormone, luteinizing hormone, estradiol, and testosterone, as well as high levels of thyroid-stimulating hormone, prolacti, and cortisol (Table 1). Peripheral blood samples were obtained, DNA was extracted from leukocytes, and the exon regions of approximately 20,000 genes in the human genome were analyzed using targeted DNA-HiSeq. The results revealed no mutations within the scope of the related diseases and no obvious chromosomal abnormalities. Magnetic resonance imaging (MRI) was performed to evaluate the morphology of the pituitary gland, but no obvious morphological changes were observed. Multiple pituitary hormone deficiency refers to the secretion disorder of multiple anterior pituitary hormones, usually including GH. A diagnosis of multiple pituitary hormone deficiency was made, and the corresponding hormone-substitution therapies (levothyroxine sodium to treat hypothyroidism and testosterone enanthate to treat hypogonadism) were initiated to prevent further deterioration of the disease.

**Table 1:** Results of laboratory examinations.

Parameter (reference range)	0 min	30 min	45 min	60 min	90 min	120 min
GH (0.02–1.5 ng/mL)	<0.02	0.03	0.05	0.05	0.02	0.06
IGF-1 (101.0–270.0 ng/mL)	25.5					
IGFBP-3 (3.30–6.60 ng/mL)	1.54					
ACTH (7.20–63.40 pg/mL)	17.2					
Cortisol (4.3–24.9 nmol/L)	111.9	105.9	102.1	115.1	123.7	156.7
PRL (72.66–407.40 mIU/L)	587.20					
TSH (0.37200–4.94000 mIU/L)	55.39					
T3 (1.35–3.15 nmol/L)	0.48					
T4 (70.0–156.0 nmol/L)	16.1					
Estradiol (40.40–161.50 pmol/L)	<37.00					
Testosterone (4.94–32.01 nmol/L)	<0.50					
FSH (0.95–11.95 IU/L)	0.90					
LH (0.57–12.07 IU/L)	0.10					

ACTH = adrenocorticotropic hormone, FSH = follicle-stimulating hormone, GH = growth hormone, IGF-1 = insulin-like growth factor-1, IGFBP-3 = insulin-like growth factor binding protein-3, LH = luteinizing hormone, PRL = prolactin, T3 = triiodothyronine, T4 = thyroxine, TSH = thyroid-stimulating hormone.

**Figure 4:**
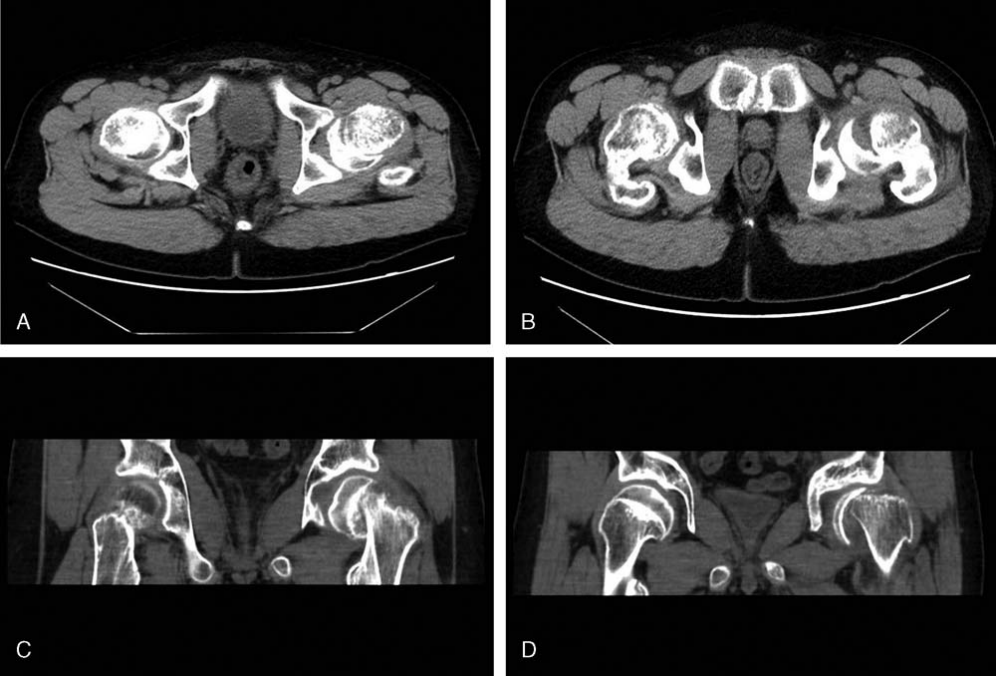
a-d. Computed tomography of both hips performed after the pain had worsened. The epiphysis has slipped backward on the left side as can be clearly observed in the coronal (A, B) and axial (C, D) scans.

After 10 days of endocrinological assessments and treatment, the patient reported a sudden worsening of hip pain. Physical examination showed that the HHS had reduced to 28, and the VAS score increased to 5. Computed tomography (CT) scanning of both hips (Fig. 4A–D) showed posterior slippage of the epiphysis relative to the metaphysis of the left hip. Therefore, the patient was transferred back to our department for further orthopedic treatment of SCFE.

**Figure 5:**
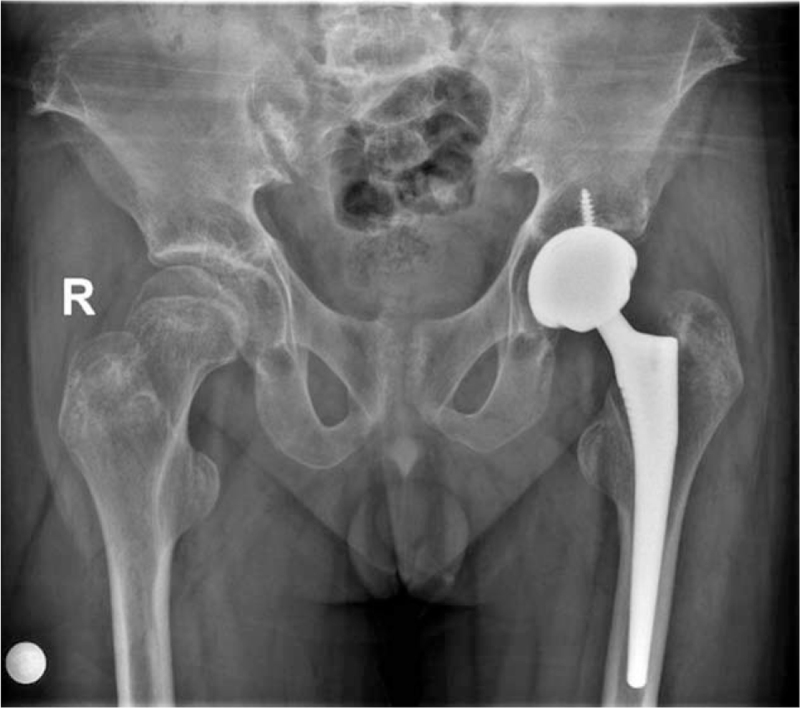
Anteroposterior pelvic radiograph was taken after total hip arthroplasty.

Since the patient was unwilling to bear the potential risk of future osteonecrosis of the femoral head and was eagerly expecting to resume work as soon as possible, we decided to perform a total hip arthroplasty (THA) for this patient after confirming the diagnosis and weighing the risks and benefits. A postoperative anteroposterior pelvic radiograph is shown in Figure 5. After the operation, the patient was instructed to undergo systematic rehabilitation exercises and to continue the previously prescribed hormone replacement therapies. After 6 months of postoperative follow-up, his gait had improved significantly, and the bilateral hip pain had been relieved (HHS: 72; VAS score: 1).

## 3 Literature review

### 3.1 Materials and methods

Relevant literature published until February 2021 was retrieved from the PubMed, Web of Science, and Embase databases. The keywords used for the searches included “slipped capital femoral epiphysis” or “SCFE” or “slipped upper femoral epiphysis” or “SUFE” and “adult,” and the search field was Title/Abstract. In addition, we screened the references of each study; therefore, articles that were not easily retrievable would not be missed. The inclusion and exclusion criteria for the literature review are listed in Table 2.

**Table 2:** Inclusion and exclusion criteria for the literature review.

Inclusion criteria	Exclusion criteria
Document type: case report or case series	Same patient was reported in different articles
Diagnosis: SCFE	Article lacks much vital information
Age at diagnosis: ≥18 y	Article not in English

SCFE = slipped capital femoral epiphysis.

### 3.2 Results

**Figure 6:**
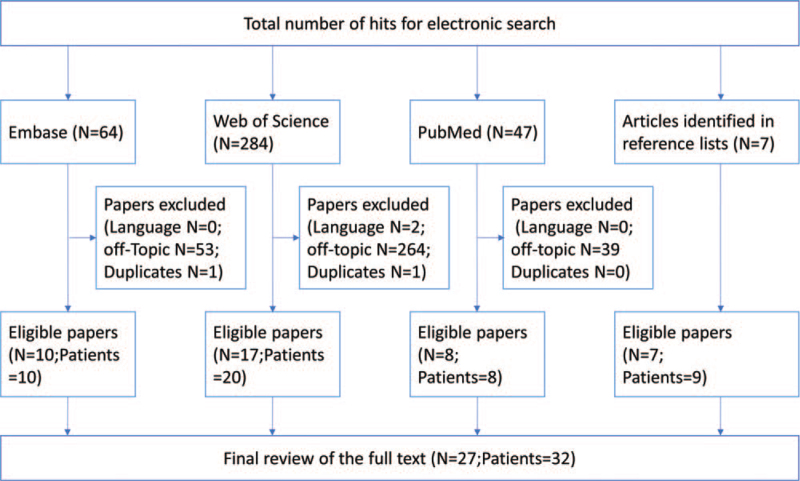
Flow diagram was presented for the retrieval and screening of the articles.

A total of 402 potentially related studies were identified through a search. After filtering out duplicate, off-topic, and non-English articles, we retrieved a total of 27 papers with 32 SCFE patients older than 18 years. The literature search process is depicted in Figure 6, and detailed information on the retrieved cases is listed in Table 3.^[4–30]^ The age of the patients at the time of onset ranged from 19 to 79 years, with a mean of 29.7 years, and of the 32 patients, 23 were men and 9 were women. Differences in the laterality of the condition were not obvious: 10 patients had bilateral slips, 12 had slips only on the left, and 10 only had slips on the right.

**Table 3:** Clinical characteristics of retrieved cases.

Author	Year	Age at diagnosis, y	Sex	Laterality	Cause	Treatment	Follow-up	Outcome
Primiano and Hughston^[1]^	1971	19	Male	NA	Hypogonadism	NA	NA	NA
Al-Aswad et al^[5]^	1978	35	Male	Right	Hypothyroidism	In situ fixation with a pin	1 y	Symptoms disappeared and all epiphyses closed
Goldman et al^[12]^	1978	19	Male	Bilateral	Renal osteodystrophy	THA	NA	NA
Hennessy and Jones^[14]^	1982	21	Male	Right	Hypothyroidism	In situ fixation with a pin	2 y	Asymptomatic, full weight-bearing gait
Montsko and de Jonge^[21]^	1995	21	Male	Bilateral	NA	NA	NA	NA
		24	Female	Right	NA	NA	NA	NA
		66	Male	Bilateral	NA	NA	NA	NA
Feydy et al^[11]^	1997	20	Male	Bilateral	Pituitary tumor	Dunn procedure	NA	NA
Moreira et al^[22]^	1998	40	Male	Right	NA	NA	NA	NA
De Silva et al^[10]^	2000	79	Male	Right	Idiopathic	In situ fixation with a screw	NA	NA
Huang and Hsu^[16]^	2007	23	Male	Right	Craniopharyngioma	In situ fixation with screws	2 y	No ipsilateral osteonecrosis or contralateral SCFE
Wang et al^[29]^	2007	27	Male	Left	Hypopituitarism, hypothyroidism, hypogonadism	In situ fixation	NA	No slippage of the contralateral hip
Nourbakhsh et al^[23]^	2008	24	Female	Bilateral	Hypothyroidism	No surgery	NA	NA
Oommen et al^[24]^	2009	29	Male	Bilateral	Hashimoto thyroiditis	In situ fixation with a screw	3 y	Could walk unaided, radiographic fusion of both epiphyses
Brady and Price^[7]^	2010	22	Male	Left	Pituitary tumor	In situ fixation of left hip with a pin, prophylactic pinning of asymptomatic right hip	2 y	Asymptomatic right hip, avascular necrosis of left femoral head, both femoral epiphyses closed, endocrinopathy symptoms practically abated
Chaganti and Tanaka^[8]^	2010	19	Male	Left	Hypogonadism	In situ fixation with a pin	18 mo	No evidence of slip in the contralateral hip
Koteles and Lewi^[19]^	2010	19	Male	Bilateral	Hypothyroidism	Open reduction and internal fixation	3 mo	Laboratory results and radiology findings tended to be normal
Hu et al^[15]^	2011	29	Male	Left	Cranio-pharyngioma	In situ fixation with a pin	18 mo	No osteonecrosis or contralateral SCF, closed bilateral proximal femoral epiphyses
Marquez et al^[20]^	2014	28	Female	Right	Hypothyroidism	In situ fixation with screws	12 mo	No slip in the contralateral proximal femoral epiphysis
Soleymanha et al^[26]^	2015	28	Female	Left	Cranio-pharyngioma	In situ fixation with a screw	4 mo	Full weight-bearing walk
Song et al^[27]^	2015	35	Male	Left	Cranio-pharyngioma	In situ fixation with a screw	2 y, 7 mo	Full weight-bearing, well-fused left femoral epiphysis, no necrosis, chondrolysis, or further slippage
		29	Male	Left	Kallmann syndrome	In situ fixation with a screw	7 years, 7 months	Both hips were asymptomatic, normal radiographic findings, no complications
		23	Male	Bilateral	Pituitary tumor	In situ fixation with a screw	8 y, 3 mo	Both hips were asymptomatic, well-fused epiphyses without further slippage or avascular necrosis
		25	Female	Bilateral	Craniopharyngioma	In situ fixation with pins	1 y after the first surgery and 2 y after the most recent procedure	Sequential slippage of the epiphysis of the right hip in the first year of follow-up after left hip surgery, well-united epiphysis without further slippage or avascular necrosis in the second year after the most recent procedure
Macia-Villa et al^[4]^	2016	47	Female	Left	Inhaled corticosteroids	THA	NA	NA
Chan et al^[9]^	2018	24	Male	Right	Pituitary tumor	Dunn procedure	NA	NA
Gupta et al^[13]^	2018	23	Male	Left	Hypopituitarism	NA	NA	NA
Assi et al^[6]^	2019	56	Female	Left	NA	No surgery	NA	NA
Huang and Hsu^[17]^	2019	29	Male	Left	Hypogonadism	Dunn procedure	6 mo	Left hip mobility gradually improved, no slip in right hip
Speirs et al^[28]^	2019	19	Male	Right	Kabuki syndrome	In situ fixation with screws	7 mo	No osteonecrosis of the femoral head, still open epiphysis, back to baseline function
Yang et al^[30]^	2019	27	Female	Bilateral	Gene mutation	In situ fixation	7 mo	Symptomatic relief
Katzen et al^[18]^	2020	21	Female	Bilateral	Hypopituitarism	In situ fixation with a screw	NA	NA
Present case	2021	37	Male	Bilateral	Hypopituitarism	THA	6 mo	Symptomatic relief

NA = not available, SCFE = slipped capital femoral epiphysis, THA = total hip arthroplasty.

### 3.3 Etiology

**Figure 7:**
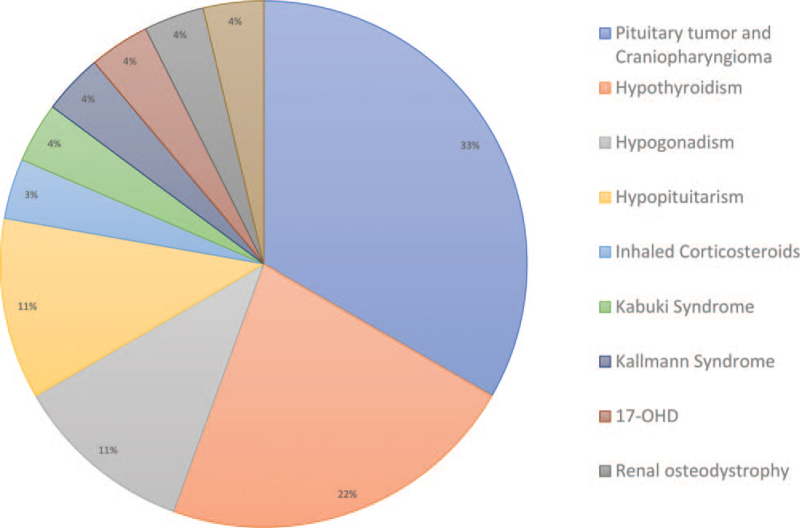
Pie chart is created for showing the potential causes and corresponding number of retrieved cases.

We noticed that most adult patients with SCFE also had an endocrine condition, such as hypopituitarism, which could have been caused by a pituitary tumor, craniopharyngioma, hypothyroidism, or hypogonadism. A total of 23 patients had endocrine disorders, including hypopituitarism, 3 patients; hypothyroidism, 7 patients; hypogonadism, 4 patients; pituitary tumor, 4 patients; craniopharyngioma, 5 patients; Kallmann syndrome, 1 patient; and 17α-hydroxylase/17,20-lyase deficiency in 1 patient (Fig. 7). Among the patients with no endocrine disorders, the etiology could be determined in 4 patients: idiopathic, Kabuki syndrome, inhaled corticosteroids, and renal osteodystrophy (n = 1 each).

### 3.4 Diagnosis

According to the medical histories of the reviewed cases, most patients experienced an episode of moderate-to-severe hip pain several days to several years before the onset of the illness, often showing abnormal growth and development. Patients with endocrine abnormalities such as short stature and stunted gonads Anteroposterior and frog-leg lateral pelvic radiographs can be used for diagnosis, and MRI and CT are greatly useful for early diagnosis and preoperative surgical planning.

### 3.5 Treatment

There are no widely agreed-upon guidelines for the treatment of adult patients with SCFE. In this study, most patients were treated using in situ fixation (n = 18), 2 patients underwent THA, 3 patients underwent the Dunn procedure, and 2 patients did not undergo any surgery. The average follow-up duration was 26 months (range, 3–99 months). Overall, the outcomes were satisfactory. There were no instances of slippage, contralateral slippage, or short- or long-term complications. In addition to the above treatments, it is important to continue treatment for accompanying endocrine diseases. Long-term hormone replacement therapy is required not only after surgery but also during the perioperative period to maintain normal metabolism.

## 4 Discussion

### 4.1 Etiology and mechanism

Endocrine disorders induce SCFE mainly through biochemical and biomechanical pathways as potential risk factors for adult SCFE. Decreased estrogen and growth spurts can reduce epiphyseal strength, and a reduction in androgen levels is not conducive to epiphyseal closure.^[31]^ In the present case report, our patient experienced a period of continuous growth in height, and his GH level decreased in adulthood. This phenomenon is recognized as growth without growth hormone and may be related to a disordered GH-IGF-1 axis and hyperinsulinemia.^[32,33]^ The delayed bone development may have been caused by GH deficiency, and the increase in body height during adulthood may be associated with hyperinsulinemia because of structural similarity between insulin and IGF-1 receptors.^[33]^ In the biomechanical pathway of disease causation, obesity increases the shear stress across the epiphysis by reducing femoral anteversion,^[34]^ and abnormal pelvic development is also an important risk factor for adult SCFE. Sankar et al^[35]^ found that the increased prevalence of SCFE may be strongly associated with greater acetabular retroversion and greater coverage area of the femoral head. Gelberman et al^[36]^ believed that decreased femoral anteversion could be specifically related to the development of SCFE. Paez et al^[37]^ suggested that morphological changes in the acetabulum of SCFE patients may be related to the etiology of their condition.

### 4.2 Diagnosis

An accurate disease classification system helps doctors choose appropriate treatments. SCFE can be classified into three types based on the course after disease onset: acute (onset <3 weeks), acute-on-chronic (acute onset based on preexisting chronic SCFE), and chronic (onset >3 weeks).^[38]^ Up to 90% of patients with acute SCFE, including the present patient, had prodromal persistent pain in the anterior thigh and hip before the onset of acute SCFE. This indicates the possibility of a chronic slip or pre-slip stage before disease onset.^[39]^ Loder et al^[40]^ proposed a classification system for SCFE based on epiphyseal stability. The slip is stable if weight bearing is possible with or without crutches; it is unstable if the patient cannot tolerate weight-bearing even with crutches. This system is helpful for evaluating epiphyseal stability and for guiding treatment. Maranho et al^[41]^ described a novel staging system for SCFE based on the anatomical structures between the epiphyseal tubercle and the metaphyseal socket. After reviewing 469 patients with SCFE, they found that their system correlated well with the severity of slip, as assessed by the Southwick head-shaft angle (*r* = 0.77, 95% CI = 0.73–0.82; *P* < .001), which could help identify patients with subtle SCFE and those in the pre-slip stage.

As a disease that mostly occurs in adolescents, it is usually difficult to accurately diagnose SCFE in adults. A comprehensive medical history and detailed physical examinations are essential for all patients. Before the onset of the slip, chronic pain is always difficult to specifically localize, and involvement of the knee joint is also possible. For adults presenting with the sudden development of unexpected secondary sexual characteristics, increase in growth velocity, appearance of central obesity, or any other growth anomalies, endocrine disorders are highly suspected, and relevant radiological examinations should be performed. Physical examination revealed limited range of motion in the hip joint and shortening deformity of the affected limb. A positive Drehmann sign, which features involuntary abduction and external rotation of the hip during hip flexion, is important evidence to confirm the diagnosis of adult SCFE.^[42]^

Radiological examination provides crucial information for the diagnosis of SCFE. Generally, slips of the epiphysis from the metaphysis can be seen on both anteroposterior and frog-leg lateral pelvic radiographs. The metaphyseal blanch sign (Steel sign), which is demonstrated on anteroposterior radiographs, is generated by the overlap of the epiphysis and metaphysis. In recent years, Song et al^[43]^ compared the acetabulotrochanteric distance between hips, and the authors believed that this approach is an easy and sensitive method to evaluate unilateral SCFE in the anteroposterior view. Early posterior slips can be detected on frog-leg lateral pelvic radiographs,^[39]^ and the Southwick head-shaft angle can be measured to evaluate the severity of the slips (normal, 12 degree; mild, <30 degree; moderate, 30–50 degree; and severe, >50 degree).^[44]^ A large Southwick angle may be an important risk factor for the development of SCFE. Lehmann et al^[45]^ performed a population-based cohort study on 2072 healthy adolescents and concluded that a greater head-shaft angle might be associated with decreased internal rotation of the hip and increased body mass index. MRI is helpful for the diagnosis of slips in the early stage or pre-slip status in symptom-free patients. Maranho et al^[46]^ retrospectively reviewed the MRI data of 71 SCFE patients treated between 2000 and 2017 and found that assessment of the peritubercle lucency sign can improve the accuracy of the early diagnoses of SCFE.

### 4.3 Treatment

In this study, the literature review showed that in most patients (n = 19), SCFE was treated with in situ fixation of the slipped femoral epiphyses, 2 patients underwent THA, and 3 patients underwent the Dunn procedure. In addition, 2 patients did not undergo any surgery, and the treatments of the remaining 6 patients were not mentioned. For patients with stable SCFE, in situ fixation with pins or screws is generally suitable. Single screw or pin fixation is more commonly performed than fixation with two or more screws or pins because the latter approach may increase the difficulty of the operation and the possibility of developing complications.^[47]^ Even with a mild change in the hip, assessment and treatment of the accompanying femoral-acetabular impingement are essential after in situ fixation.^[48]^ There is controversy over whether the contralateral femoral head should be fixed at the same time for patients with unilateral SCFE. According to a retrospective study by Woelfle et al^[49]^, prophylactic contralateral fixation of SCFE is a reliable procedure that does not generate major complications. Epiphysiodesis with bone graft is conventionally indicated for stable slips; however, an unacceptable re-slippage rate was reported by Adamczyk et al.^[50]^

Compared with stable SCFE, unstable SCFE is more difficult to manage and is associated with more severe complications, including avascular necrosis (AVN) of the femoral head. It is vital to protect the blood supply of the epiphysis during treatment to prevent AVN of the femoral head. The alignment of the proximal femur should be optimally restored to overcome the potential risk of femoral-acetabular impingement development. Recently, the modified Dunn procedure has been increasingly recommended for the treatment of unstable and severe SCFE because of its advantages. Lerch et al^[51]^ performed a retrospective study involving 46 patients with severe SCFE treated with the modified Dunn procedure and found low incidence rates of postoperative AVN (5%) and osteoarthritis (2%) of the hip. A case series published by Elmarghany et al^[52]^ in 2017 enrolled 30 patients who were treated with the modified Dunn procedure and followed up for an average of 14.5 months; the slip angle of the femoral head was corrected to a mean of 5.6 ± 8.2 degree, and the normal proximal femoral anatomical structure was restored.^[52]^ For adults with SCFE, THA is also an appropriate choice because the risk of re-slippage no longer exists, and joint function can be restored immediately after the operation, allowing the early resumption of their lives. A retrospective study conducted by Francesco et al^[52,53]^ demonstrated that THA has the advantages of a low complication rate and better restoration of leg length. However, some authors, such as Larson et al^[54]^, believe that SCFE patients undergoing THA have a moderately high revision rate.

## 5 Conclusions

Adult SCFE is a rare disease that can be caused by a variety of conditions that are not fully understood or are beyond our knowledge. We reported an adult SCFE case, at the same time, reviewed the characteristics of adult SCFE and the advances in its diagnosis and treatment in publications. Most adult patients with SCFE have preexisting endocrine disorders that require attention. Only by carefully evaluating the stability of the epiphysis and fully considering postoperative complications can an appropriate treatment be selected. Information about the diagnosis and management of adult SCFE remains insufficient, and more relevant studies are expected to provide a better understanding of this disease.

## Author contributions

**Conceptualization:** Tong Liu, Jianlin Zuo.

**Data curation:** Zhixin Niu, Jinshuo Tang, Shenghao Xu.

**Formal analysis:** Zhixin Niu.

**Funding acquisition:** Tong Liu.

**Investigation:** Jinshuo Tang, Xianyue Shen, Shenghao Xu, Zhongsheng Zhou.

**Methodology:** Zhixin Niu.

**Project administration:** Tong Liu.

**Resources:** Tong Liu.

**Software:** Xianyue Shen.

**Supervision:** Jinshuo Tang.

**Validation:** Xianyue Shen, Tong Liu.

**Visualization:** Zhongsheng Zhou.

**Writing – original draft:** Zhixin Niu.

**Writing – review & editing:** Jinshuo Tang, Tong Liu, Jianlin Zuo.
